# Impact of glutathione peroxidase 4 on cell proliferation, angiogenesis and cytokine production in hepatocellular carcinoma

**DOI:** 10.18632/oncotarget.24300

**Published:** 2018-01-22

**Authors:** Nataliya Rohr-Udilova, Eva Bauer, Gerald Timelthaler, Robert Eferl, Klaus Stolze, Matthias Pinter, Martha Seif, Hubert Hayden, Thomas Reiberger, Rolf Schulte-Hermann, Markus Peck-Radosavljevic, Dagmar Stoiber, Michael Trauner

**Affiliations:** ^1^ Division of Gastroenterology and Hepatology, Internal Medicine III, Medical University of Vienna, Vienna, Austria; ^2^ Ludwig Boltzmann Institute for Cancer Research, Vienna, Austria; ^3^ Institute of Cancer Research, Internal Medicine I, Medical University of Vienna, Vienna, Austria; ^4^ Department of Life Sciences, Veterinary University of Vienna, Vienna, Austria; ^5^ Institute of Animal Nutrition and Functional Plan Compounds, Department of Farm Animals and Veterinary Public Health, Veterinary University of Vienna, Vienna, Austria; ^6^ Clinic Klagenfurth, Division of Gastroenterology and Hepatology, Klagenfurt am Woerthersee, Austria; ^7^ Institute of Pharmacology, Center for Physiology and Pharmacology, Medical University of Vienna, Vienna, Austria

**Keywords:** phospholipid glutathione peroxidase, free radicals, cell proliferation, angiogenesis, hepatocellular carcinoma

## Abstract

Insufficient supplementation with the micronutrient selenium and persistent hepatic inflammation predispose to hepatocellular carcinoma (HCC). Inflammation-associated reactive oxygen species attack membrane lipids and form lipid hydroperoxides able to propagate oxidative hepatic damage. Selenium-containing enzyme glutathione peroxidase 4 (GPx4) antagonizes this damage by reducing lipid hydroperoxides to respective hydroxides. However, the role of GPx4 in HCC remains elusive.

We generated two human HCC cell lines with stable overexpression of GPx4, performed xenotransplantation into NOD.Cg-Prkdc^scid^Il2rg^tm1Wjl^/SzJ (NSG) host mice and characterized the tumors formed. The experimental data were verified using gene expression data from two independent HCC patient cohorts.

GPx4 overexpression protected from oxidative stress and reduced intracellular free radical level. GPx4-overexpressing cells displayed impaired tumor growth, reduced proliferation, altered angiogenesis and decreased expression of clinically relevant cytokine interleukin-8 and C-reactive protein. Moreover, GPx4 overexpression impaired migration of endothelial cells *in vitro*, and enhanced expression of thrombospondin 1, an endogenous inhibitor of angiogenesis. In patients, GPx4 expression in tumors positively correlated with survival and was linked to pathways which regulate cell proliferation, motility, tissue remodelling, immune response and M1 macrophage polarization. The patient data largely confirmed experimental findings especially in a subclass of poor prognosis tumors with high proliferation.

GPx4 suppresses formation and progression of HCC by inhibition of angiogenesis and tumor cell proliferation as well as by immune-mediated mechanisms. Modification of GPx4 expression may represent a novel tool for HCC prevention or treatment.

## INTRODUCTION

Hepatocellular carcinoma (HCC) is the second leading cause of cancer-related deaths worldwide [1]. HCC epidemiology is currently changing due to the availability of highly-effective antiviral drugs against HBV and HCV infections as well as due to increasing incidence of other cancer-predisposing liver diseases such as alcoholic and non-alcoholic steatohepatitis (ASH, NASH) and non-alcoholic fatty liver disease (NAFLD) [2].

Chronic hepatic inflammation is a common feature of cancer-predisposing liver diseases and favours HCC development [3]. There is a broad consensus that oxidative stress mediated by reactive oxygen species (ROS) plays a pivotal role [4]. Oxidative stress may not only interfere with cellular homeostasis and metabolism but also contributes to lipotoxicity. ROS initiate peroxidation of polyunsaturated fatty acids resulting in formation of fatty acid hydroperoxides, as shown for heating of dietary oils or inflammation [5]. Fatty acid hydroperoxides can undergo Fenton-type decomposition thus increasing intracellular radical level, propagating lipid peroxidation and favouring mutagenesis and regenerative cell proliferation.

We have previously found that linoleic acid hydroperoxide apparently contributes to NASH and HCC and that selenium antagonises the effects in HCC [6, 7]. In line, selenium inversely correlates with tumor size in Austrian HCC patients [7]. Moreover, epidemiological studies revealed an increased HCC risk in patients with low systemic levels of selenium [8–10] whereas selenium supplementation decreased the risk [10].

Selenium as selenocysteine is present in the catalytic centre of antioxidative enzymes. Five of the 25 characterized human selenoproteins belong to a family of glutathione peroxidases which reduce hydroperoxides to the respective alcohols by oxidation of two glutathione molecules [11]. Hydroperoxides are cytotoxic at higher concentrations while lower concentrations are essential for intra- and extracellular signalling and adequate immune response [12].

Glutathione peroxidase 4 (GPx4) is essential for life - at least in mice [13]. The unique function of GPx4 consists in reduction of lipid hydroperoxides within membranes thus preventing formation of secondary radicals [14] and inhibiting further lipid peroxidation [11]. The expression and activity of GPx4 depends on selenium [15]. The liver is particularly sensitive to moderate selenium deficiency since other organs such as brain and reproductive system take up selenium with higher priority [15].

In a previous mechanistic study, we found inverse relations between lipid hydroperoxides and selenium levels in hepatocarcinogenesis [7]. Inhibition of GPx4 expression by siRNA in HCC cells increased formation of cytokines VEGF and IL-8 [7] both of which are clinically relevant adverse prognostic factors in HCC patients [16, 17].

Complementary to our previous study [7], we now assessed the effects of GPx4 overexpression in HCC and analysed correlations between GPx4 and cancer patient survival. Further, we have re-evaluated gene expression microarray data from 140 human HCCs and conducted pathway analyses. Finally, a new system biology approach allowed us to analyze and to compare the composition of infiltrating immune cells in human HCC tumors stratified according to the intratumoral GPx4 levels.

Data from humans confirmed the experimental data obtained in cell culture and animal xenografts. Results of molecular, biochemical and histological studies clearly support the protective role of GPx4 in liver carcinogenesis. Our results indicate that GPx4 inhibits cell proliferation, migration, angiogenesis and immune cell infiltration. An increase of GPx4 activity may represent a novel tool for HCC treatment or prevention.

## RESULTS

### *In vitro* characterisation of HCC cells overexpressing GPx4

**Figure 1:**
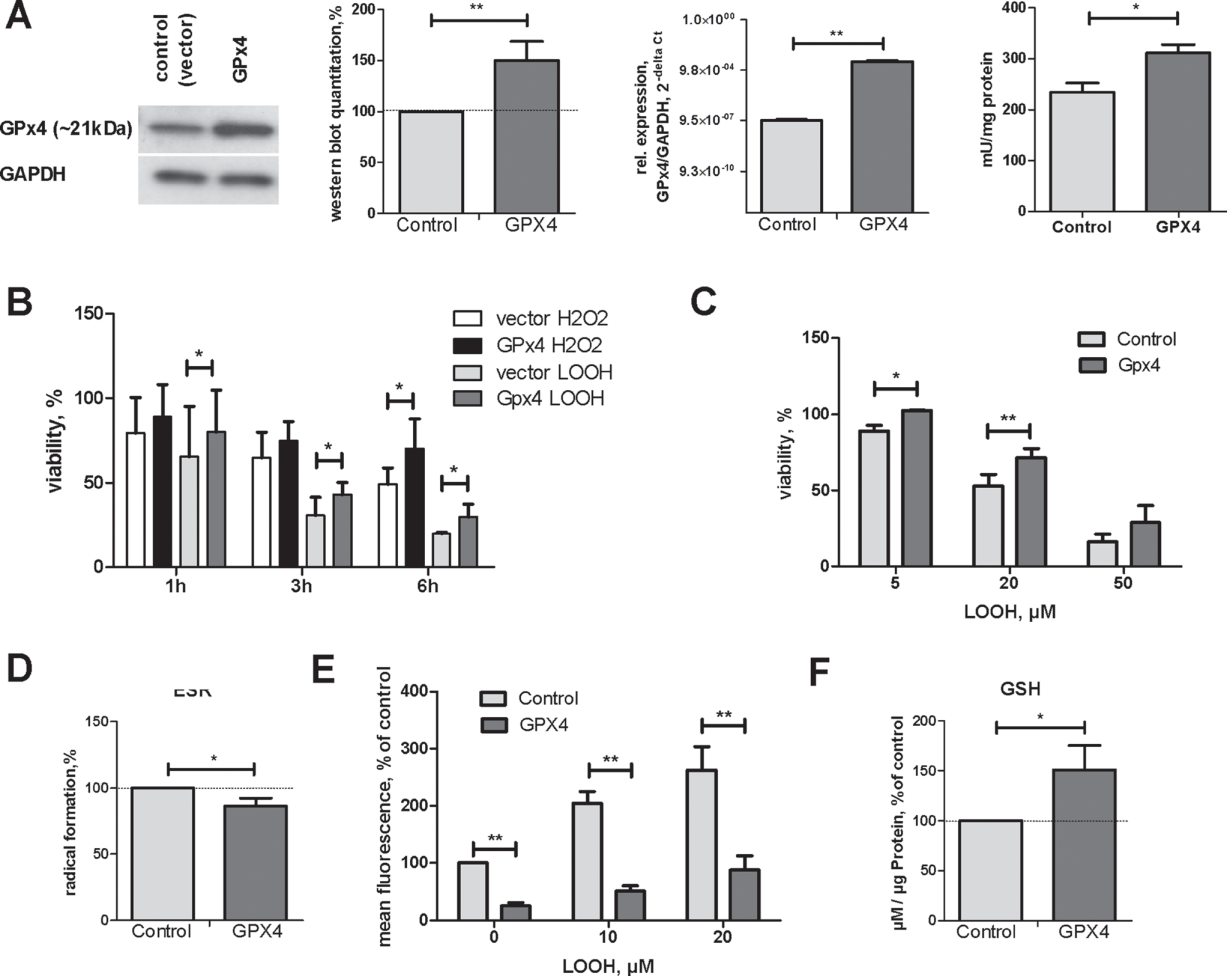
Characterisation of HCC-3 cells overexpressing GPx4 (**A**) A representative western blot, quantification summary, mRNA levels and activity in GPx4-transfected HCC-3 cells (Gpx4); control (vector) , ^**^p < 0.01; ^*^p < 0.05. (**B**) HCC-3 control (vector) or GPx4 overexpressing cells were incubated with 200 µM H_2_O_2_ or with 30 µM LOOH for the indicated time and viable cells quantified with neutral red. (**C**) HCC-3 were incubated with LOOH for 24 h and viability was assessed. (**D**) HCC-3 cells were mixed with CMH and analysed by ESR spectroscopy as described in supplementary Materials and Methods. Three independent cell preparations were measured in three repeats each and summarized. (**E**) 10^5^ HCC-3 cells were incubated with LOOH for 2h in the presence of DCFH. Intracellular mean fluorescence was quantified by flow cytometry. (**F**) Intracellular GSH concentration in control and GPx4 overexpressing HCC-3 cells was determined as described in Materials and Methods.

To explore GPx4 functions in HCC, we introduced the porcine GPx4 gene into the human HCC-3 cell line. Western blotting, real-time RT-PCR and activity measurements confirmed GPx4 overexpression (Figure 1A). GPx4 overexpressing cells were more resistant to cell death induced by hydrogen peroxide and peroxidized linoleic acid (LOOH, Figure 1B, 1C). Notably, GPx4 overexpression reduced intracellular radical level as demonstrated by two independent methods (Figure 1D, 1E). In line with diminished radical stress, we found increased intracellular glutathione concentrations in cells overexpressing GPx4 (Figure 1F).

**Figure 2:**
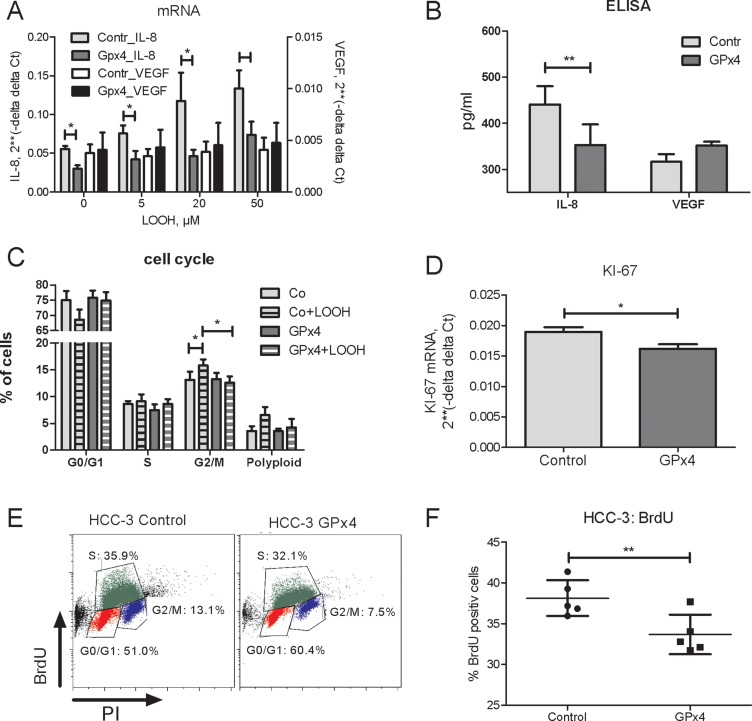
Impact of Gpx4 overexpression on gene expression and cell cycle progression in HCC-3 cells (**A**) GPx 4 overexpressing (GPx4) or control (vector) HCC-3 cells were treated by LOOH for 3 hours and IL-8 mRNA and VEGF mRNA were analysed by real-time RT-PCR. (**B**) Protein concentrations of IL-8 and VEGF in supernatants of HCC-3 cells (6 h). (**C**) Cell cycle progression in HCC cells treated by 20µM LOOH or by vehicle for 3 hours. (**D**) HCC-3 cells (3 × 10^5^/well) were seeded into 6 well plates and KI-67 mRNA was analysed by real-time RT-PCR 24 h later. (**E**) Impact of GPx4 overexpression in HCC-3 cells on BrdU incorporation: representative plot for HCC-3 control and HCC-3 GPx4 overexpressing cells. (**F**) Quantitation summary of BrdU incorporation. ^*^p < 0.05; ^**^p < 0.01.

Down-regulation of GPx4 in hepatocarcinoma cells induces VEGF and IL-8 [7], the two cytokines associated with poor HCC prognosis [16, 17]. Complementary, overexpression of GPx4 in HCC tumor cells lowered IL-8 levels at base line and upon LOOH treatment (Figure 2A, 2B). In contrast, VEGF mRNA and protein levels remained constant (Figure 2A, 2B).

We have previously shown that LOOH induce HCC cell cycle progression [18]. Consistently, GPx4 overexpression reduced the LOOH-induced increase of cells in G2/M phase (Figure 2C). Furthermore, GPx4 inhibited HCC cell proliferation as indicated by a decreased mRNA of the proliferation marker KI-67 (Figure 2D) as well as by BrdU incorporation (Figure 2E, 2F). In addition, supernatants from GPx4 overexpressing HCC-3 cells treated by LOOH showed a decreased capacity to induce the migration of HUVECs (Supplementary Figure 2).

### Effect of GPx4 overexpression on *in vivo* tumor growth in a xenograft model

**Figure 3:**
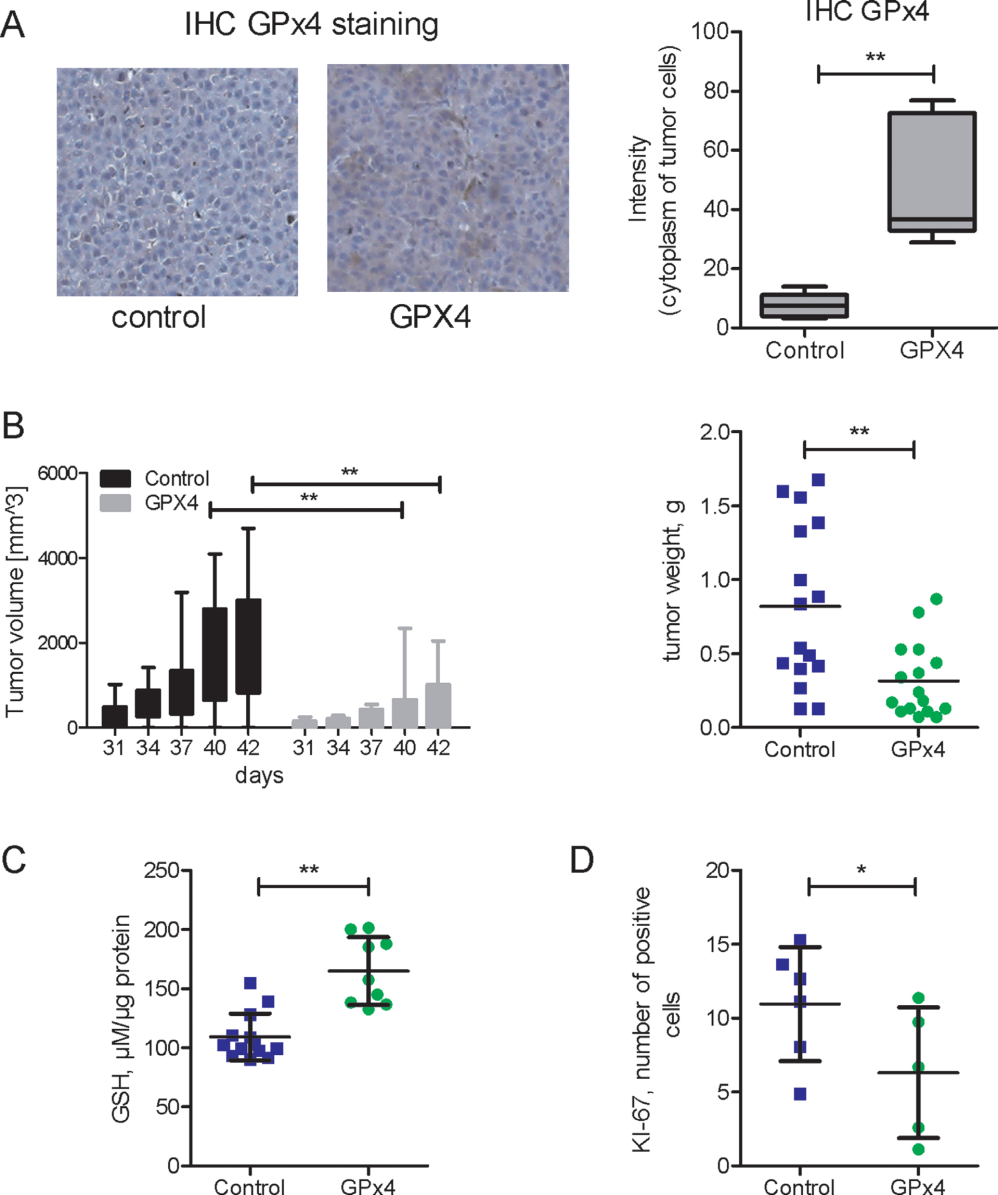
*In vivo* growth of xenograft HCC tumors overexpressing GP x 4 (**A**) IHC GP x 4 staining and histomorphometric analysis of GP x 4 staining intensity in xenograft tumors originating from HCC-3 cells, either control-vector or overexpressing GP x 4 (GP x 4). (*n* = 5 per group, ^**^p < 0.01) (**B**) Growth kinetics (days) and final weight of xenograft tumors (*n* = 16 in each group, ^**^p < 0.01). For subsequent investigations, the tumors were harvested at the end of experiment on day 42. (**C**) Glutathione content in lysates of GP x 4 overexpressing and control (vector) tumors (*n* = 9–13, *p* < 0.01). (**D**) Histomorphometric analysis of KI-67-positive cells in xenograft tumors (*n* = 5–6, *p* < 0.05).

*In vivo* effects of GPx4 overexpression were addressed in a xenograft NSG mouse model. GPx4 overexpression persisted in xenograft tumors (Figure 3A). GPx4-overexpressing HCC-3 derived tumors grew slower as compared to vector transfected cells and exhibited reduced final tumor weight (Figure 3B). In line with cell culture *in vitro* experiments (Figure 1F, Figure 2D–2F), we found elevated glutathione (GSH) levels (Figure 3C) as well as reduced cell proliferation (Figure 3D) in GPx4-overexpressing tumors.

### GPx4 overexpression and angiogenesis in HCC-3 tumors

**Figure 4:**
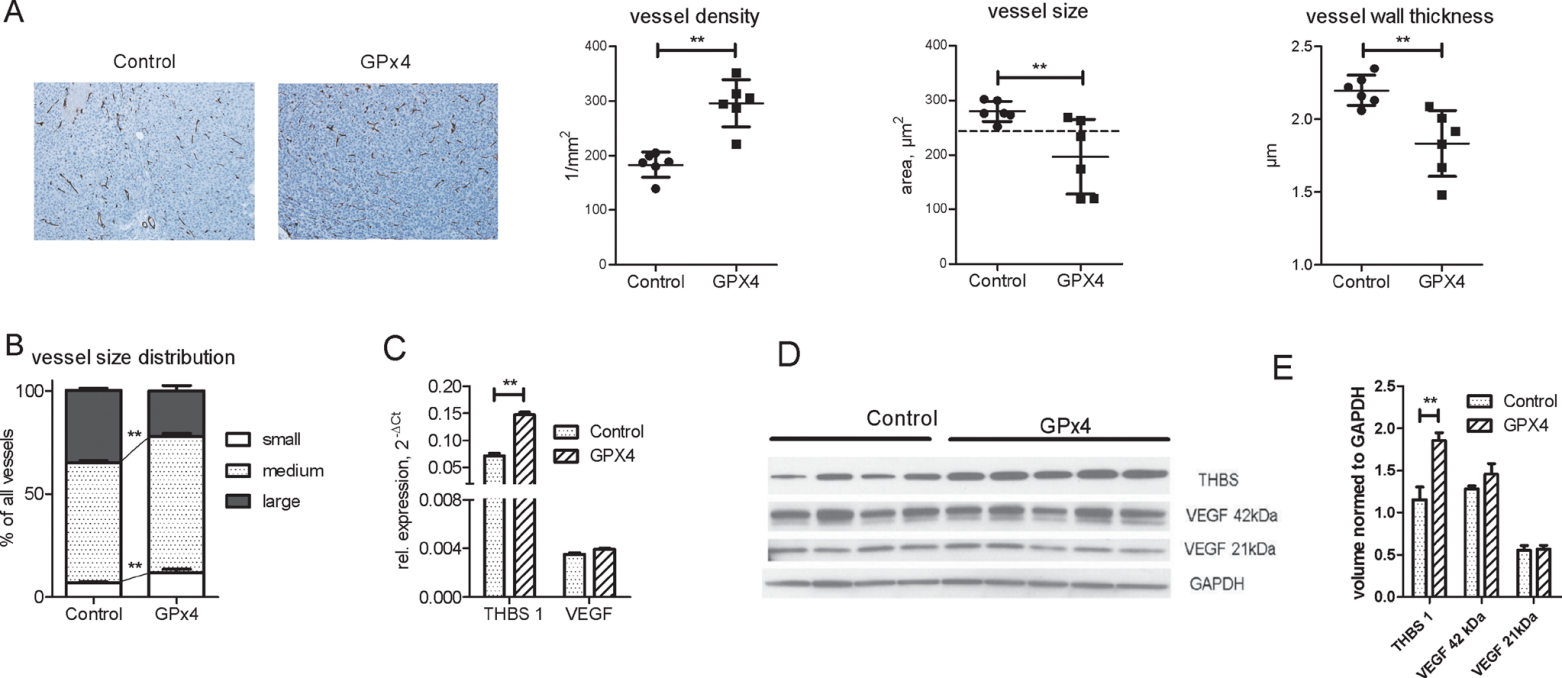
Angiogenesis in control and GP x 4 overexpressing xenograft tumors (**A**) A representative endomucin vessel staining and histomorphometric analysis of vessel density, size and wall thickness in GPx4 overexpressing and control xenograft tumors, *n* = 6 per group, *p* < 0.01. (**B**) Impact of GPx4 overexpression on vessel size distribution: mean percentage of large, medium and small vessels was calculated for each group, *n* = 6 in each group, ^**^p < 0.01. Real-time RT-PCR (**C**), Western blot (**D**) and western blot quantification of THBS1 and VEGF in lysates of tumors with and without GPx4 overexpression. ^**^p < 0.01.

Xenograft tumors overexpressing GPx4 exhibited an increased vessel density but decreased vessel diameter and wall thickness (Figure 4A). Small and medium vessels prevailed in tumors with GPx4 overexpression (Figure 4B).

Interestingly, vessel wall thickness correlated with the percentage of large vessels (Supplementary Figure 3A). Moreover, other vessel parameters as vessel size, vessel wall thickness and percentage of large vessels also correlated with tumor volume (Supplementary Figure 3B). These results imply that larger vessels with thicker wall support tumor growth more efficiently while GPx4 overexpression constrains growth of large tumor vessels.

To investigate potential regulators of the observed angiogenic pattern, we focused on thrombospondin 1 (THBS1), an endogenous inhibitor of angiogenesis [19]. Indeed, GPx4 overexpression increased THBS1 expression in tumors and cultured cells (Figure 4C–4E, Supplementary Figure 4A), whereas VEGF expression remained constant (Figure 4C–4E).

### GPx4 overexpression and formation of IL-8 and C-reactive proteins

**Figure 5:**
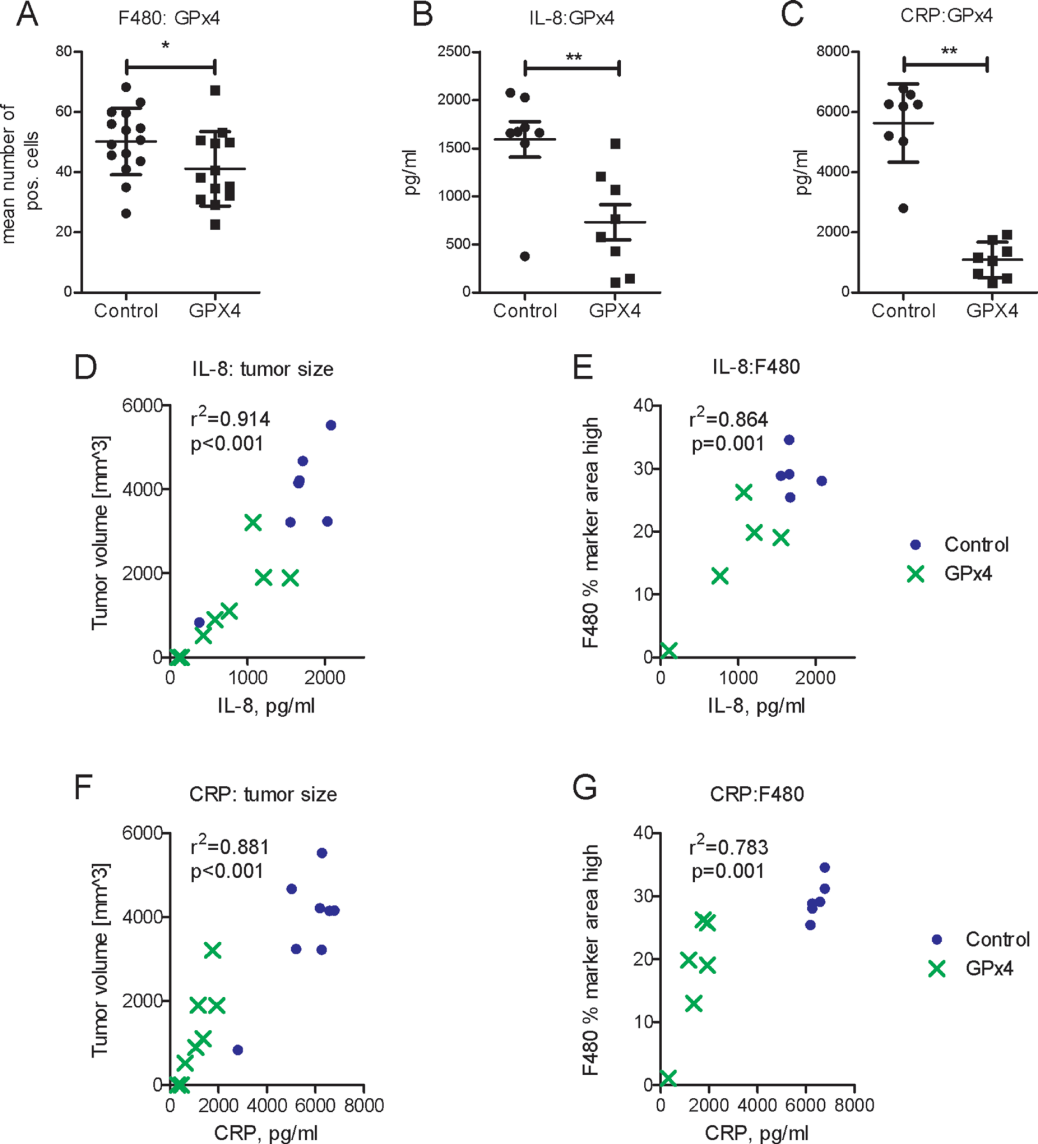
Impact of GPx4 overexpression in tumor cells on macrophage infiltration, IL-8 and C-reactive protein (**A**) Histomorphometric analysis of F4/80 positive cells representing macrophages in control (Co) and GPx4 overexpressing tumors (GPx4), *n* = 13–15, ^*^p < 0.05. Serum protein concentrations of IL-8 (**B**) and C-reactive protein (CRP) (panel **C**) in animals bearing GPX4- overexpressing or control tumors ^*^p < 0.05, ^**^p < 0.01, *n* = 8. Correlations (r^2^ Pearson) of serum IL-8 with tumor size (**D**) and macrophage infiltration (**E**) in xenograft model, *n* = 8. Correlations of serum CRP with tumor size (**F**) and macrophage infiltration (**G**) in xenograft model, *n* = 8.

Tumor-associated macrophages promote tumor angiogenesis [20]. We found that GPx4 overexpression reduced the density of F4/80 positive macrophages in xenografts (Figure 5A).

Importantly, IL-8 exerts chemotactic activity on lymphocytes and neutrophils [20]. Neutrophils, in turn, recruit macrophages thus stimulating tumor growth [21]. In addition to IL-8, C-reactive protein (CRP) also facilitates macrophage infiltration and has clinically prognostic value in HCC [22]. GPx4 overexpression reduced the concentrations of both, serum IL-8 and CRP proteins in the xenograft model (Figure 5B, 5C). Both, serum IL-8 and CRP, correlated with tumor size and with macrophage infiltration (Figure 5D–5G). Decreased CRP expression was confirmed *in vitro* in GPx4 overexpressing HCC-3 cells (Supplementary Figure 4B).

The key results obtained with GPx4 overexpressing HCC-3 cells were confirmed in the second transfected human HCC cell line, Huh7. Tumors originating from GPx4 overexpressing Huh7 cells were smaller, had higher glutathione content, increased THBS1 and decreased CRP expression as well as altered angiogenic pattern (Supplementary Figure 5).

### Confirmation of GPx4 effects in human HCC

To check the relevance of the above experimental findings for humans, we investigated associations between survival of cancer patients and mRNA levels of GPx4 and 23 other selenoproteins. We applied the recently established *Prediction of Clinical Outcome from Genomic Profiles (PRECOG)* database which combines survival and microarray gene expression data from ∼18000 cancer patients [23].

GPx4 is the only selenoprotein which is consistently associated with a prolonged survival in cancer including HCC (Supplementary Table 1, Supplementary Figure 6).

To further explore the role of GPx4 in human HCC and to strengthen the significance of our experimental findings, we re-evaluated microarray gene expression from two independent HCC patient cohorts: i) *n* = 60 [24] and ii) *n* = 80 [25]. Natural variations of GPx4 expression allowed us to stratify the patients according to their GPx4 level. Using median as a cut-off value, we separated the “low GPx4” and the “high GPx4” patient groups and compared the mean expression values of THBS1, KI-67, CRP, VEGF, IL-8 and human F4/80 homolog EMR1 between the groups.

Since HCC tumors are extremely heterogeneous, molecular HCC subclasses S1, S2 and S3 specify patients with similar deregulated pathways and similar prognosis. In particular, patients with S1 and S2 subclass tumors have poor prognosis as compared to S3 subclass [25].

**Figure 6:**
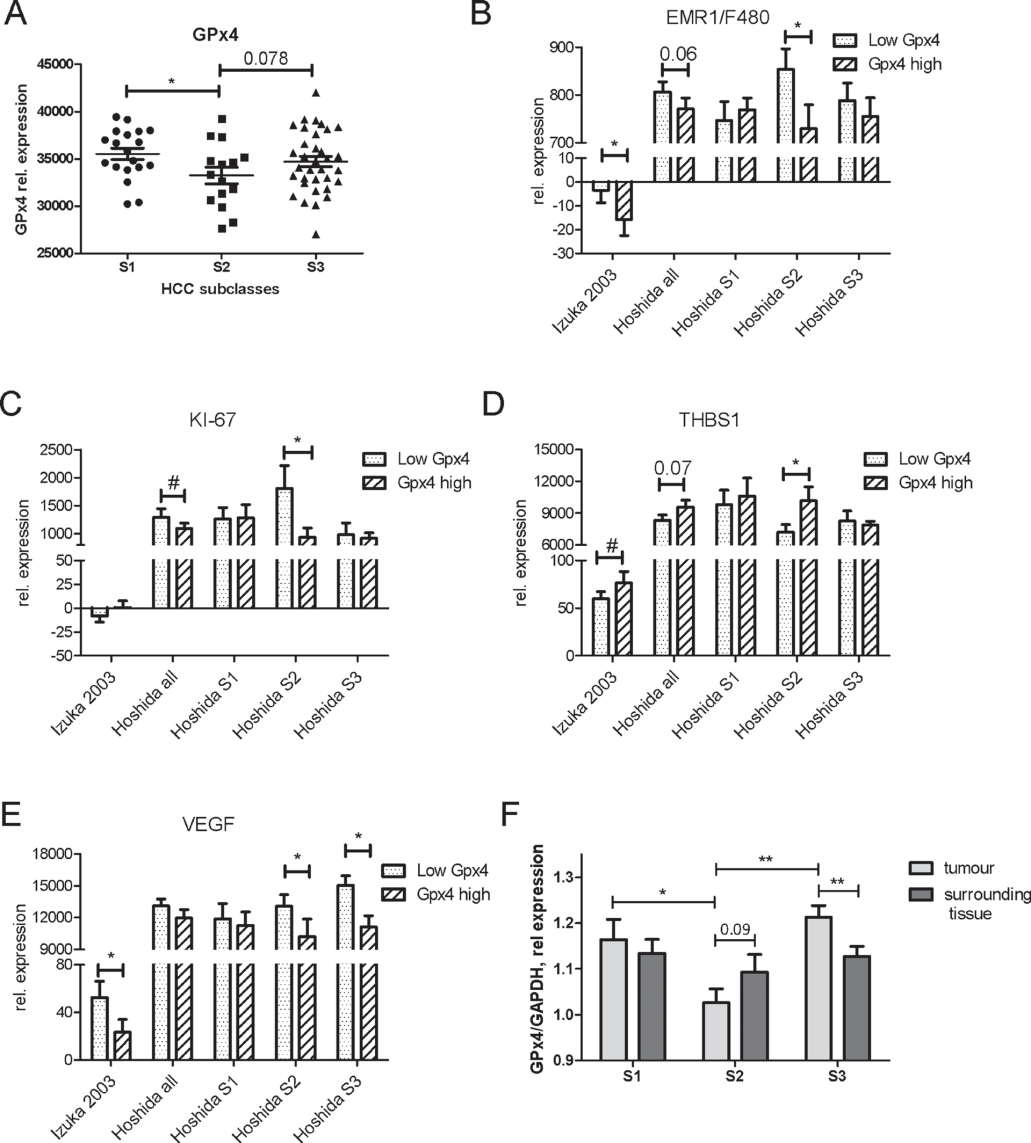
Interactions of GPx4 with gene expression in human HCC patients Re-evaluation of gene expression microarray data in tumor tissues from two cohorts of human HCC [23, 24]. The cohort of Hoshida was subdivided into molecular subclasses S1, S2 and S3. (**A**) Expression levels of GP x 4 in tumor tissues of patients from S1, S2 and S3 HCC subclasses. ^*^p < 0.05 S1 vs S2. The patients were stratified according to the normalized GPx4 expression levels. Mean gene expression values of EMR1/F4/80 (**B**), KI-67 (**C**), THBS1 (**D**), and VEGF (**E**) were compared between “Low GPx4” or “High Gpx4” patient groups. ^*^p < 0.05; ^**^p < 0.01 Mann-Whitney one-tailed *t*-test; # variances are different at *p* < 0.05. (**F**) Relative GPx4 expression was compared between HCC tumor tissues and surrounding tissues from the patients with different molecular subclasses of HCC in the Hoshida cohort. ^*^p < 0.05, ^**^p < 0.01 two sides unpaired *t*-test, *n* = 18 for S1, *n* = 9 for S2, *n* = 26 for S3 group.

To assess the role of GPx4 in different molecular backgrounds, we additionally compared the differences between low and high GPx4 expression for each molecular subclass. Interestingly, GPx4 expression was lower in the proliferation subclass S2 than in S1 (*p* < 0.05) and S3 (trend, *p* = 0.078) (Figure 6A). The “high GPx4” group within the S2 subclass showed a decreased expression of EMR1/F4/80, KI-67 and VEGF as well as an increased expression of THBS1 as compared to the “low GPx4” group (Figure 6B–6E). The whole cohorts showed less pronounced but similar changes. Down-regulation of CRP and IL-8 by experimental GPx4 overexpression was recapitulated in the “high GPx4” patient group and particularly within the S1 subclass (Supplementary Figure 7A, 7B).

We have further compared relative GPx4 expression between tumor tissue and surrounding tissue in HCC patients with different molecular subclasses. As Figure 6F shows, GPx4 level in surrounding tissue differs from that in tumor. In particular, S3 subclass with a better prognosis showed higher GPx4 level in tumors than in corresponding surrounding tissues. In contrast, S2 subclass showed an opposite trend: higher GPx4 level in surrounding tissues than in tumors. There were no differences in GPx4 level between surrounding and tumor tissues in S1 subclass.

Thus, data from human HCC and specifically from S2 molecular HCC subclass largely support experimental findings on GPx4 impact on cell proliferation, cytokine release, angiogenesis and immune cell infiltration.

### Pathways differentially regulated in HCCs from patients with low and high tumor GPx4 levels

To analyze the pathways differentially regulated by GPx4 in human HCCs, we stratified patients according to their GPx4 expression levels in tumor, defined quartiles with the high and the low GPx4 expression and applied Ingenuity Pathway Analysis. The results supported our *in vitro* data and revealed pathways involved in cell migration, adhesion and immune response (Table 1, Supplementary Table 2).

**Table 1:** Differentially regulated canonical pathways between the lowest and the highest quartile of GPx4 expression in tumor tissue of HCC patients

Ingenuity Canonical Pathways	-log(p-value)	Ratio	Molecules
Hepatic Fibrosis / Hepatic Stellate Cell Activation	9,44E00	4,92E-02	COL6A1, COL6A3, COL6A2, CCL21, IGFBP5, MYH11, COL4A2, COL15A1, COL3A1
Granulocyte Adhesion and Diapedesis	5,46E00	3,39E-02	CCL21, CXCL12, THY1, CCL14, CCL19, SELPLG
Agranulocyte Adhesion and Diapedesis	5,3E00	3,17E-02	CCL21, CXCL12, CCL14, MYH11, CCL19, SELPLG
Primary Immunodeficiency Signaling	3,61E00	5,77E-02	IL7R, IGHG3, IGKC
B Cell Development	2,54E00	5,88E-02	IL7R, IGKC
Atherosclerosis Signaling	2,51E00	2,42E-02	CXCL12, COL3A1, SELPLG
Hematopoiesis from Pluripotent Stem Cells	2,2E00	3,92E-02	IGHG3, IGKC
ILK Signaling	2,03E00	1,61E-02	VIM, MYH11, TMSB10/TMSB4X
Leukocyte Extravasation Signaling	1,95E00	1,52E-02	CXCL12, THY1, SELPLG
Actin Cytoskeleton Signaling	1,85E00	1,38E-02	F2R, MYH11, TMSB10/TMSB4X
Systemic Lupus Erythematosus Signaling	1,83E00	1,36E-02	IGHG3, IGKC, C7
TR/RXR Activation	1,77E00	2,35E-02	UCP2, COL6A3
Factors Promoting Cardiogenesis in Vertebrates	1,71E00	2,17E-02	TCF4, MEF2C

A number of pathways differentially regulated by GPx4 in human HCC deals with immune cell and their transmigration and include diapedesis of granulocytes (neutrophils, basophils and eosinophils) and agranulocytes ( lymphocytes and monocytes) as well as B cell development and primary immunodeficiency signaling (Table 1). Other two differentially regulated pathways – actin cytoskeleton signaling and integrin-linked kinase (ILK) signaling – further support the involvement of GPx4 in the regulation of cell movement. Finally, pathways involved in hepatic stellate cells activation and fibrosis as well as in thyroid hormone receptor / retinoid X receptor signaling (TR/RXR) also differed between the HCC patients from the highest and the lowest GPx4 expression quartiles. Thus, impact of GPx4 on immune cells in HCC deserves further attention.

### Potential effects of GPx4 on immune cells in HCC

**Figure 7:**
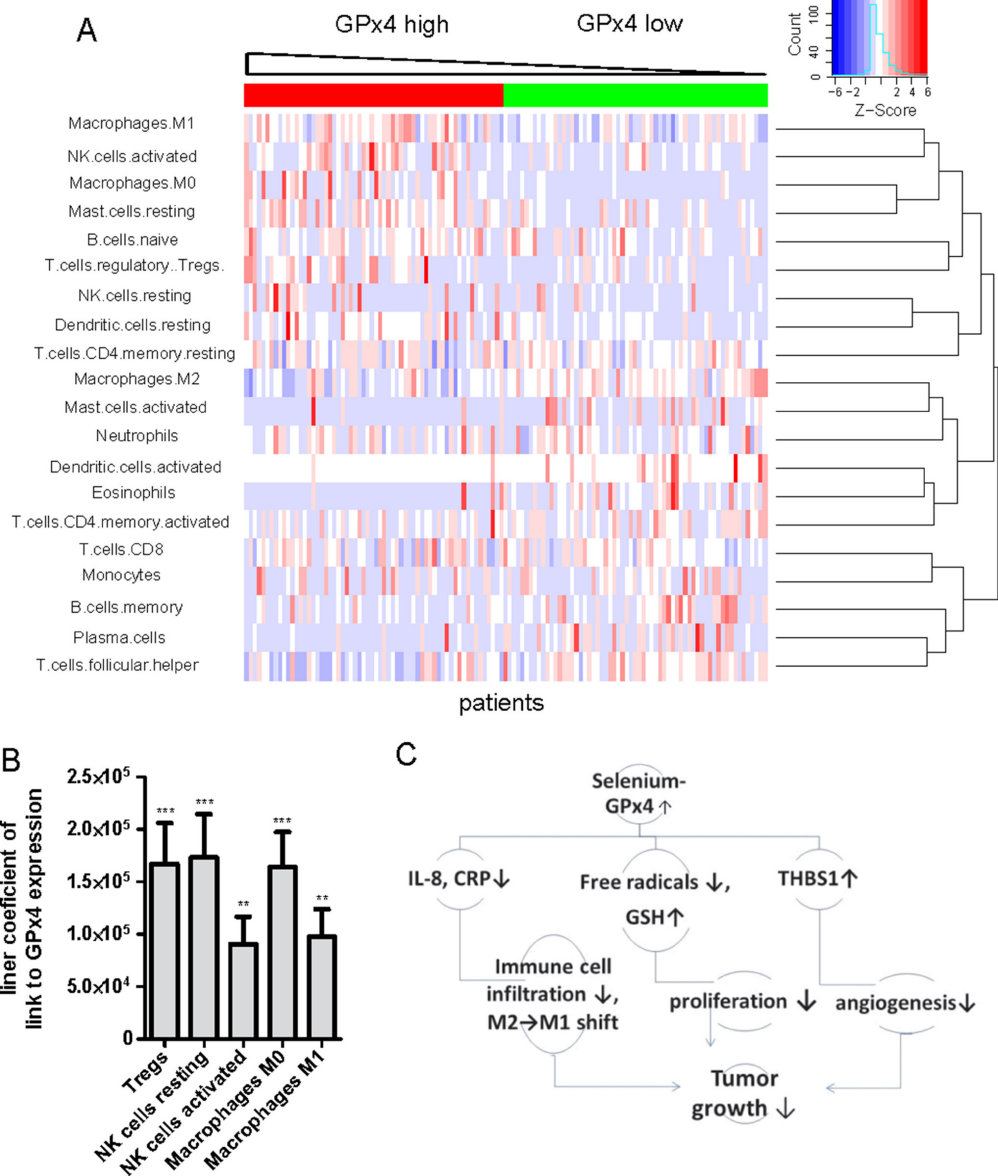
GPx4 expression level and profiles of tumor infiltrating immune cells in human HCC (**A**) Heat map of the infiltrating immune cells composition as determined by CIBERSORT method in relation to GP x 4 expression level within tumors. Each column represents one patient. (**B**) Linear coefficients of the regression analysis between amounts of immune cells types and GP x 4 expression levels in patients. ^**^p < 0.01; ^***^p < 0.0001 (**C**) Suggested mechanisms of protection by selenoenzyme GP x 4 in HCC.

Next, we used a novel CIBERSORT approach to compare the immune cell composition between the low/high GPx4 patient groups. These groups have been previously defined in the preceeding Result sections. CIBERSORT immune cell profile for each patient has been calculated from microarray gene expression data. The results are shown in Figure 7A. We have also compared the mean values for each immune cell type between the low and high GPX4 patient groups (Supplementary Table 3). We found that M0 macrophages, regulatory T cells and activated NK cells were strongly increased whereas eosinophils, γδ T cells and activated dendritic cells were depleted in high GPx4 tumors. High GPx4 expression was also associated with a shift from M2 toward M1 macrophages (Supplementary Table 3) characteristic for tumor suppression [20]. We could recapitulate chemoattraction of neutrophils by IL-8 in our dataset (Supplementary Table 4). However, the percentage of neutrophils, CD4^+^ memory resting T cells, CD8^+^ T cells and monocytes remained constant between the low / high GPx4 groups.

Taking into account expression levels EMR1/F480 in immune cells [23] and the percentage of each immune cell type, we identified eosinophil and M2 macrophage populations are the main contributors to the reduced EMR1/F4/80 levels in “high GPx4” patients (Supplementary Table 5). Thus, higher expression of GPx4 is associated with a distinct profile of infiltrating immune cells in human HCCs.

## DISCUSSION

The role of selenoenzyme GPx4 in HCC formation is currently unknown. To our knowledge, this is the first study which provides convincing evidences in favor of tumor suppressive function of GPx4 in HCC specifically in context of highly upregulated proliferation. In particularly, we found that overexpression of GPx4 in HCC cells reduced free radical levels, increased GSH and decreased proliferation (Figure 7C). Moreover, GPx4 reduced formation of the HCC-promoting cytokine IL-8, inhibited cell cycle progression and reduced cell migration after pro-oxidative challenge with linoleic acid hydroperoxide. Importantly, analysis of gene expression data from human HCC tumors recapitulated most of our experimental *in vitro* and *in vivo* findings.

Epidemiologically, selenium is highly relevant for HCC protection in humans in areas with low soil selenium as Europe and China [8–10]. Recently, a cohort within the EPIC (European Prospective Investigation into Cancer and Nutrition) study showed that decreased plasma selenium is associated with increased risk of HCC [8]. These human data agree with protective role of selenium in progression and promotion phases of experimental HCC [26]. As our mechanistic data suggests, GPx4 significantly contributes to HCC protection by selenium, even if other selenoproteins as SELP, DIO1 and TXNRD2 show similar positive association with cancer survival (Supplementary Table 1) and may also be involved.

GPx4 level in human tissues seems to be tightly regulated suggesting that only a narrow GPx4 range is physiological. Distribution frequency of naturally possible expression levels in different individuals gives us an idea which GPx4 levels are common and thus physiological. Indeed, the distribution width of GPx4 expression is narrow when compared with all genes (NCBI Aceview, Supplementary Figure 8A). For narrow distribution, even relatively small shift in GPx4 expression level may already bring it close to its physiological limit (Supplementary Figure 8A). A narrow physiological distribution of GPx4 expression could be an explanation for the relative small changes in protein and activity increase even using a strong CMV promoter in our experiments. Relatively small changes in GPx4 mRNA levels we have observed in patients are also consistent with the tight GPx4 regulation (Supplementary Figure 8B).

The net impact of GPx4 on HCC biology depends on the molecular context which differs between the molecular HCC subclasses S1, S2 and S3 selected on the basis of similar gene expression patterns [25]. We found that GPx4 insufficiency is particularly relevant for S2 subclass of tumors which is characterized by increased proliferation and WNT-pathway activation.

In contrast to S2, molecular subclasses S1 and S3 might provide a different context for the role of GPx4. For example, accumulation of GPx4 in tumor tissue as compared to surrounding tissue has been described in hepatitis C-related liver tumors [27]. This finding could be retraced in our data for S3 molecular subclass (Figure 6F), which predominates in hepatitis C-related tumors [25]. In addition, even if GPx4 mRNA and protein accumulate, it does not necessarily increase GPx4 activity as the function may still be lost.

### Impact of Gpx4 on intracellular radicals, redox status and cell proliferation

GPx4 plays apparently a fundamental role in the regulation of radical homeostasis and regulates a specific type of cell death called ferroptosis [28]. We have shown that GPx4 overexpression in HCC cells decreased intracellular radicals which, in turn, induce cell proliferation [29]. As GSH can directly scavenge radicals, high glutathione content inhibited tumor cell proliferation in rat HCC model [30] and might thus contribute to the anti-proliferative GPx4 effects.

*In vivo*, transplanted GPx4-overexpressing HCC cells formed smaller tumors which displayed reduced tumor cell proliferation and reduced macrophage density.

In agreement with our findings in HCC, GPx4 inhibits cell cycle progression and decrease growth of xenograft tumors also in non-HCC cancer cells [31, 32]. GPx4 per se attenuates tolerance towards oxidative stress [33, 34] or synergizes with the lipophilic radical scavenger vitamin E in hepatocytes [35].

Lipid hydroperoxides are substrates of GPx4 and originate from both enzymatic (lipoxygenases, cyclooxygenases) and non-enzymatic (free radicals) lipid peroxidation. Polyunsaturated fatty acids–e.g. linoleic acid - are particularly prone to radical-induced peroxidation. Peroxidized linoleic acid (LOOH) is crucial for pathogenesis of both, non-alcoholic steatohepatitis (NASH) and HCC [7, 36] and provides a relevant context for protective GPx4 activity.

### Impact of GPx4 overexpression on tumor angiogenesis and infiltrating immune cells

Overexpression of GPx4 in HCC cells reduced pro-angiogenic IL-8 and enhanced anti-angiogenic THBS1 levels and altered angiogenic pattern in tumors. GPx4-overespressign tumors showed reduced percentage of large vessels and thinner vessel walls. In line with our findings here, GPx4 deficiency in tumors increased angiogenesis [37].

Here, we discovered that GPx4 consistently increases THBS1 levels *in vitro* and *in vivo,* just in agreement with human HCC data showing the same positive correlation. THBS1 is a potent endogenous inhibitor of angiogenesis, blocks nitric oxide-driven vascular smooth muscle cells relaxation and is involved in HCC [19]. THBS1 upregulation together with the impaired migration of endothelial cells might be the mechanisms how GPx4 inhibits tumor angiogenesis. Indeed, THBS1 overexpression led to a decreased average vessel size and a reduced percentage of large vessels [38] – all the features also observed in GPx4 - overexpressing tumors.

We observed a clear linear correlation between the percentage of large vessels and vessel wall thickness (Supplementary Figure 3A) that may reflect retained physiological normality of tumor vessels and indicate the intact pericyte coverage [39].

We have showed that high GPx4 expression in human HCC is associated with a distinct composition of infiltrating immune cells: γδT-cells, eosinophils and activated dendritic cells were reduced, whereas M1-macrophages, activated NK-cells and regulatory T-cells were increased.

Such a composition of infiltrating immune cells might be beneficial. Indeed, γδT cells favour development of HCC [40], although tumor suppressive functions have been described in other cancer types. In addition, M1-macrophages are anti-tumorigenic and positively correlate with survival of HCC patients [41].

GPx4 overexpression and increased GSH levels may affect not only immune cell composition but also function. In particular, higher levels of thiols can improve the cytotoxic activity of CD8^+^ T cells [42]. These immune-cellular mechanisms might also contribute to the inverse correlation between glutathione concentration and tumor size observed in our study.

Collectively, these data suggest that protection by GPx4 in hepatocarcinogenesis is mediated – at least in part – by the improved immune response.

### Outlook for clinical implications

The suggested protective role of GPx4 in hepatocarcinogenesis opens novel preventive and therapeutic strategies. GPx4 mimetics, selenium prodrugs and oral selenium substitution are all potential strategies to increase hepatic GPx4 activity in patients at risk and in those with established HCC. However, genetic polymorphism that affects GPx4 protein synthesis [43] has to be considered.

Previous SELECT study on cancer prevention by selenium did not show any benefits [44]. However, this study involved patients from the USA where soil selenium concentration is higher than in Europe and seems not to be a limiting factor. Consequently, systemic selenium concentrations in patients from the SELECT study (∼130 ng/ml) were higher as compared to patients at increased HCC risk from the European EPIC study (∼90 ng/ml) [8]. Therefore, studies on HCC prevention in patients with low selenium are required.

Our data suggest that patients with the S2 molecular subclass of HCC might particularly benefit from therapeutic interventions that increase GPx4 expression. Therefore, molecular classification of HCC and assessment of serum/plasma selenium levels, hepatic GPx4 levels, CRP/IL-8 and radical stress markers [36] should help to identify target populations for therapeutic GPx4 modulation. Interventional enhancement of GPx4 levels might also increase tumor-infiltrating M1 macrophages as an adjuvant tool to enhance immune therapy responses. However, these concepts require further confirmation in future clinical studies.

## MATERIALS AND METHODS

### Cellular experiments

Human HCC-3 cell line (kind gift of Prof. B. Grasl-Kraupp) has been established from the tumor of an Austrian patient [45]; Huh7 were purchased (ATCC). Cells were kept under standard tissue culture conditions and if not otherwise indicated supplied with 50nM sodium selenite. Synthesized LOOH [14] was dispersed by sonication into serum free medium containing 1mg/ml fatty acid free BSA. pcDNA3-GPx4 (pocine) construct was a kind gift of Prof. R. Brigelius-Flohé [46] and was transfected into human HCC-3 and Huh7 hepatocarcinoma cells. After growth selection with geneticin, we generated a stable transfected cell lines with GPx4 overexpression as confirmed by real-time PCR, western blotting, and activity measurements. Control cells were transfected with empty pcDNA3 vector and subjected to the same selection procedure. Cells with the same passage number were used for comparison between control and GPx4 overexpression and no more than 25 passages were conducted. The cells were regularly checked for mycoplasma contaminations.

2′,7′-dichlorofluorescin diacetate (DCFH) fluorescence, glutathione concentration and cell cycle were analysed as described earlier [18, 47].

### Animal xenograft models

5 × 10^6^ HCC-3 or 1 × 10^6^ Huh7 cells (in 200 µl of a 1:1vol/vol PBS/matrigel) were injected subcutaneously into each flank of 13-15 weeks old male NSG (NOD *scid* γ) mice and formed tumors within 4-7 weeks. The experiments were performed with agreement of the local ethical committee.

### Patient data

Microarray gene expression and survival data of cancer patient from the established *Prediction of Clinical Outcome from Genomic Profiles (PRECOG)* database [23] were used. PRECOG contains survival data from ∼18000 patients diagnosed with 39 distinct malignancies including HCC.

Affimetrix gene expression datasets from two HCC patient cohorts have been re-evaluated: sixty patients from the cohort of Iizuka [24] and eighty patients from the cohort of Hoshida [25].

### Free radical detection by electron spin resonance (ESR)

10^6^ cells/ml suspended in PBS containing 100µM DTPA were mixed with 10 µl of 20 mM CMH and analyzed using Bruker ESR spectrometer ESP300e. 100 µM DTPA did not affect viability of HCC-3 cells (Supplementary Figure 1). See Supplementary Methods for more details.

### Quantification of infiltrating immune cells

The quantification of infiltrating immune cells in human HCCs has been performed by CIBERSORT method [23, 48]. CIBERSORT allows enumeration of 22 immune cell types by applying signatures from ∼500 marker genes to microarray gene expression data from tumors to quantify the contribution of each cell type [48]. The method has been previously validated by flow cytometry [48].

### Immunohistochemistry

F4/80 and KI-67 staining was applied to analyse macrophage infiltration and cell proliferation respectively. To visualise vessel formation, HCC tissues were stained for endomucin (Endomucin-Ab, eBiosciences, No. 14-5851-81, 1:500) according to [49] and digitalised using a Pannoramic Midi Slide Scanner (3Dhistech, Budapest, Hungary). Quantitative characteristics of vessel morphology were obtained by histomorphometric analyses of digitized slides using the Tissue Studio^®^ software (Definiens, Munich, Germany).

### Real time RT-PCR and western blotting

mRNA was isolated, converted to cDNA and analysed by real time RT-PCR Taqman System as published earlier [7] using the following primers: Hs00173626_m1 for VEGF, Hs00174103_m1 for IL-8, Hs01591589_m1 for GPx2 and Hs00157812_m1 for Gpx4, Hs00357041_m1 for CRP, Mm04207460_m1 for CXCL1 (Applied Biosystems). Western blotting was described earlier [7, 50]. GPx4 polyclonal antibody (Cayman Chemicals No.10005258) detected both porcine and human GPx4.

### ELISA protein measurements

ELISA kits for human serum Quantikine VEGF (R&D Systems, Abingdon, UK), IL-8 (BenderMedSystems GmbH, Vienna, Austria), CRP (Abcam, Cambrodge, UK) and THBS1 (R&D Systems, Abingdon, UK) were all used according to the manufacturers’ instructions.

### Statistics

If not otherwise indicated, all cellular data were obtained from *n* = 5 independent experiments. Data are expressed as mean ± SD, and statistical differences were determined using ANOVA with significance accepted at *p* < 0.05.

## SUPPLEMENTARY MATERIALS FIGURES AND TABLES



## Abbreviations

CMH: 1-Hydroxy-3-methoxycarbonyl-2,2,5,5-tetramethylpyrrolidine . HCl
CRP: C-reactive protein
CXCL1: chemokine (C-X-C motif) ligand 1
DCFH: 2′,7′-dichlorofluorescin diacetate
DTPA: Diethylenetriaminepentaacetic acid
EMR1: adhesion G protein-coupled receptor E1 (human)
F4/80: adhesion G protein-coupled receptor E1 (mouse)
GPx: glutathione peroxidase
GSH: glutathione (reduced)
IL-8: interleukin 8
FCS: fetal calf serum
HCC: hepatocellular carcinoma
LDH: lactate dehydrogenase
LH: linoleic acid
LOOH: linoleic acid hydroperoxides
ROS: reactive oxygen species
THBS1: thrombospondin 1
VEGF: vascular endothelial growth factor
NSG: NOD scid γ (NOD.Cg-Prkdcscid Il2rgtm1Wjl/SzJ)
