# Supplementary material for: Impact of glutathione peroxidase 4 on cell proliferation, angiogenesis and cytokine production in hepatocellular carcinoma
Source: Oncotarget. 2018 Jan 22;9(11):10054–68. doi: 10.18632/oncotarget.24300 (PMC5839371; doi:10.18632/oncotarget.24300)
Supplement: Supplementary file 1 [file oncotarget-09-10054-s001.pdf]

Impact of glutathione peroxidase 4 on cell proliferation, angiogenesis and cytokine production in hepatocellular carcinoma

SUPPLEMENTARY MATERIALS

Free radical detection by electron spin resonance (ESR)

10^6 cells/ml suspended in PBS containing 100 µM DTPA were mixed with 10 µl of 20 mM CMH and analyzed using Bruker ESR spectrometer ESP300e equipped with a TM^{110} microwave cavity and a 500µl quartz flat cell was used, operating at X-band frequencies (~9.8 GHz) with 100 kHz modulation frequency with the following settings: 3484 G center field, 80G sweep width; 0.54 G modulation amplitude; 81.92 ms time constant; 81,92 ms conversion time; 1024 data points; 20 mW microwave power; 5 scans accumulated; gain: 1×10^3. The signal amplitude has been normalized to the protein concentration. Three experiments have been performed with each preparation; three independent preparations were summarized.

HUVEC migration assay

HUVECs (a kind gift of B. Winter, Medical University of Vienna, Austria) were grown in EGM-2 Bullet Kit Media (Lonza) under standard tissue culture conditions and used at passages between 4 and 8. Human umbilical vein endothelial cells (HUVECs) were seeded at a density of 2×10^4 cells per well into the upper insert of a Boyden-Chamber equipped with 8µm Nuclepore Track-Etch Membrane (Whatman, Clifton, NJ, USA) coated with rat tail collagen (BD Biosciences). The lower chamber was filled with conditioned cell culture media collected from HCC-3/Vector or HCC-3/GPx4 cells previously treated with 20 µM linoleic acid (LH) or 20 µM linoleic acid hydroperoxide (LOOH). The cells were incubated for 6 h at 37ºC (5% CO2). Migration activity was evaluated as the mean number of migrated cells in 5 random fields (40x) per well. The experiment was performed four times.

Human protein atlas and GTEx datasets

The Genotype-Tissue Expression (GTEx) Project was supported by the Common Fund of the Office of the Director of the National Institutes of Health, and by NCI, NHGRI, NHLBI, NIDA, NIMH, and NINDS. The data used for the analyses described in this manuscript were obtained from GTEx Portal on 09/01/17.

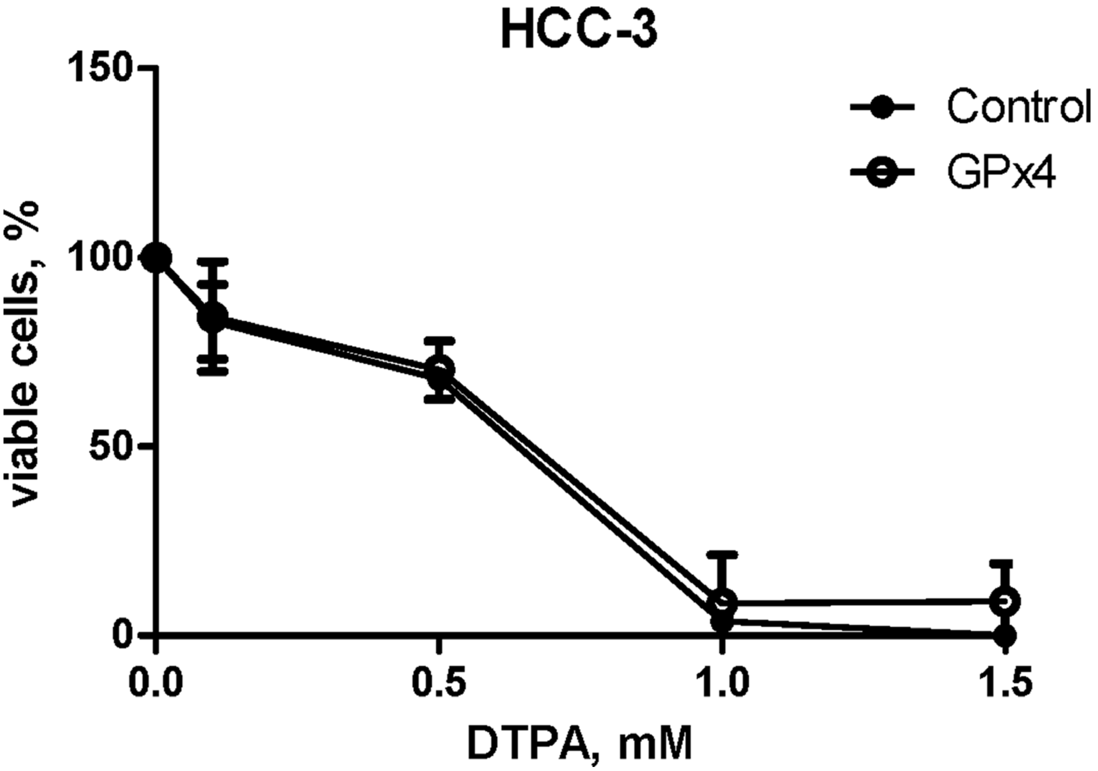

Supplementary Figure 1: Impact of iron chelator di-ethylenetriamine pentaacetic acid (DTPA) on HCC-3 cell viability. DTPA at indicated concentrations was added for 24 h to HCC-3 control or GPx4 overexpressing cells previously grown in 24-well plates. Viable cells were quantified by neutral red assay as described in Materials and Methods section. Each experiment was performed in triplicates and the results of two independent experiments are summarized.

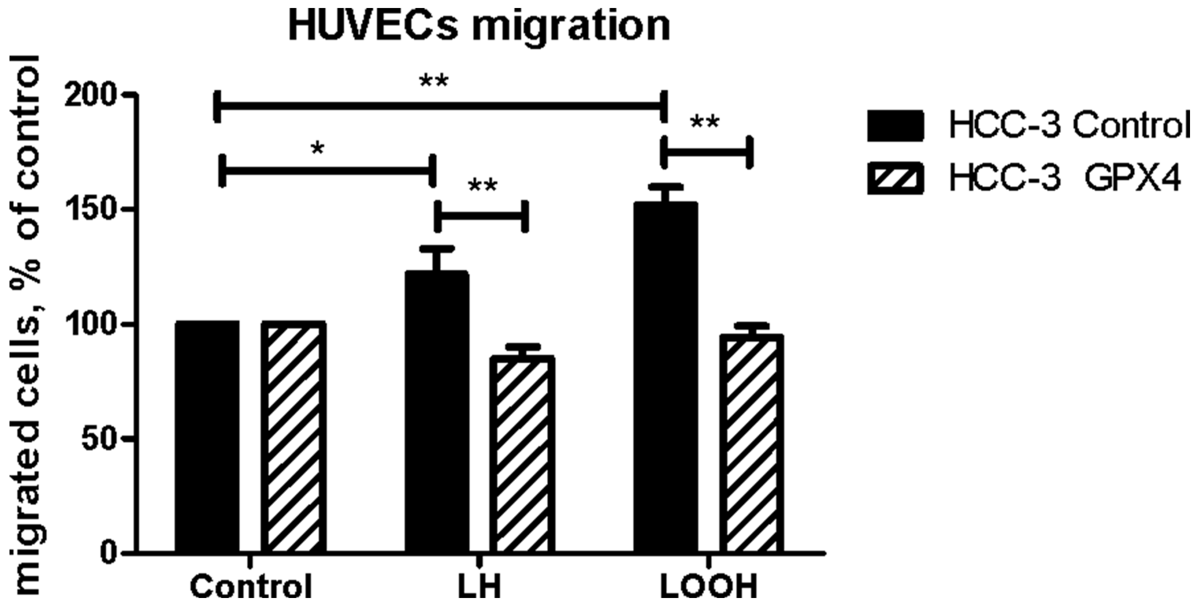

Supplementary Figure 2: Impact of GPx4 on cell migration. Migration of HUVECs towards supernatants harvested from HCC-3 GPx4 overexpressing and control cells treated by 20µM linoleic acid (LH), 20µM peroxidized linoleic acid (LOOH) or vehicle for 3h. $^{*}p < 0.05$, $^{**}p < 0.01$, $n = 4$.

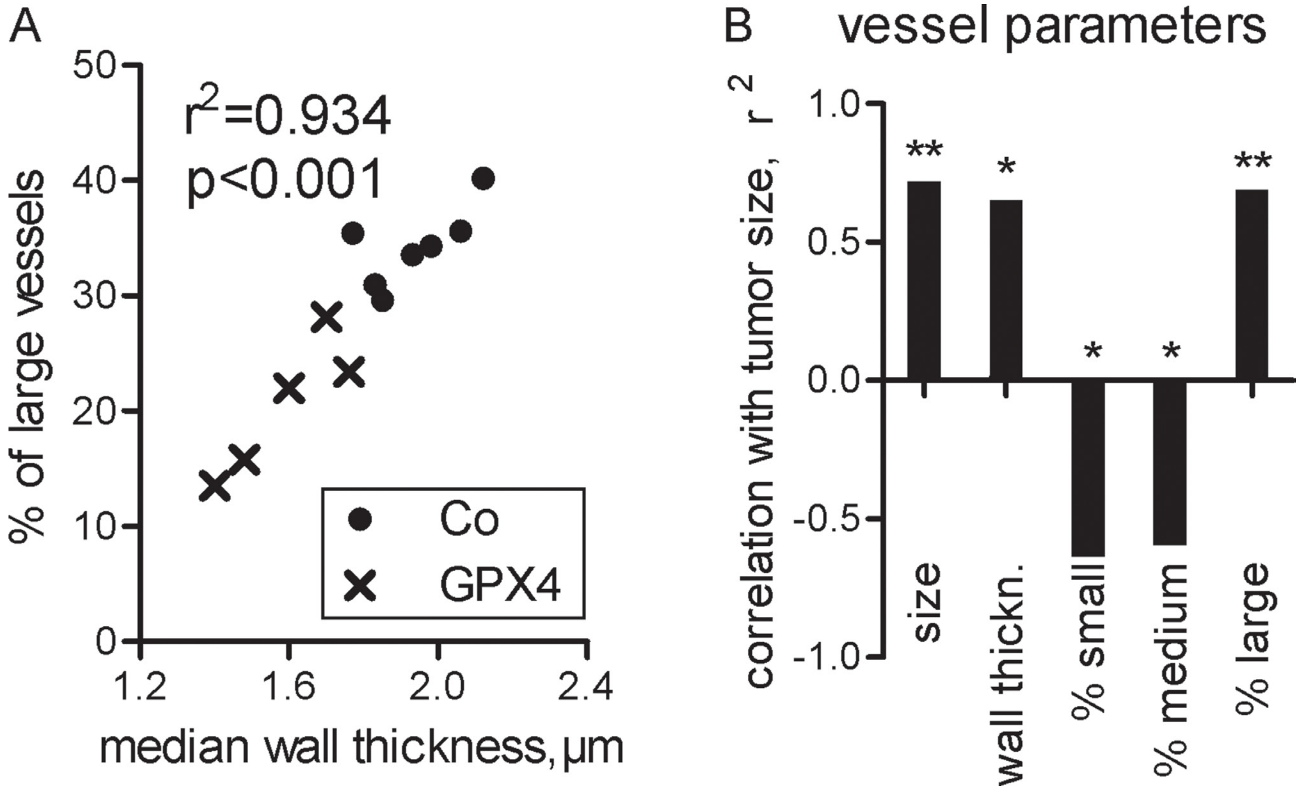

Supplementary Figure 3: Correlation between vessel parameters in tumors derived from GPx4 overexpressing HCC-3 cells in NSG xenografts. Vessel parameters were determined by histomorphomeric analysis of vessel IHC endumucin staining in xenograft tumors. (**A**) Correlation between the median wall thickness and the percentage of large vessels within the tumors. Each dot represent one tumor, every value was calculated as a mean for all vessels stained within the tumor. (**B**) Correlation coefficients (r^2 Pearson) between final tumor size and vessel parameters, which were calculated as mean values for all vessels stained. N = 10-16 tumors, $^{*}p < 0.05$. $^{**}p < 0.01$.

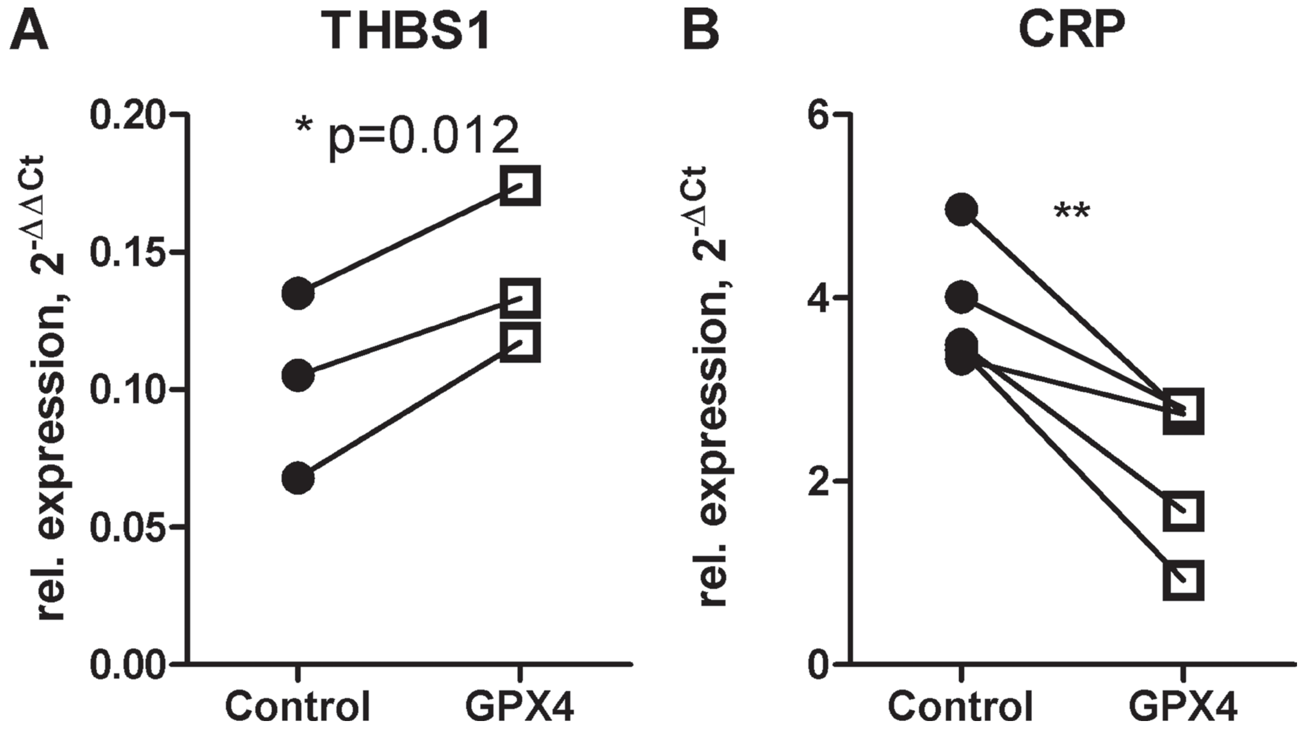

Supplementary Figure 4: Effect of GPx4 overexpression in HCC-3 cells on thrombospondin 1 and C-reactive protein. HCC-3 control or GPx4 overexpressing cells were seeded into 6-well plates and grown in full medium at 5% O2 until they reach 70-80% confluence. (**A**) Thrombospondin 1 mRNA was analysed in triplicates by real-time RT-PCR and normalized to GAPDH as a house keeping gene. The results of three independent experiments are summarized. $P < 0.05$, paired one-tailed *t*-test. (**B**) CRP mRNA was analysed in triplicates by real-time RT-PCR and normalized to GAPDH as a house keeping gene. The results of five independent experiments are summarized. $^{**}p < 0.01$, paired one-tailed *t*-test.

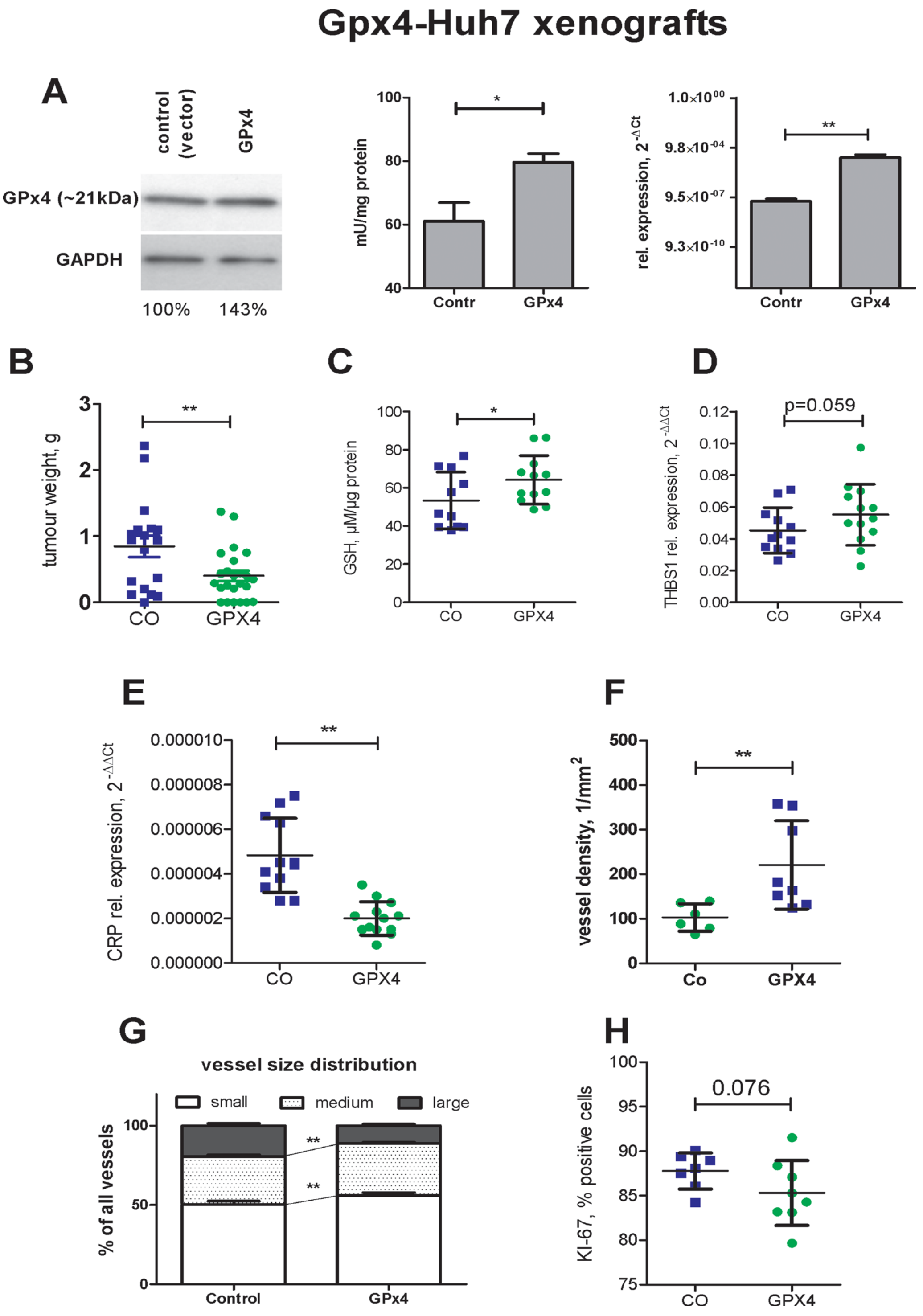

Supplementary Figure 5: *In vivo* growth of xenograft tumors originating from Huh7 control or GPx4 overexpressing cells. Huh7 cells were stably transfected with porcine GPx4 plasmid and 1 × 106 cells were diluted into 200 µl 1:1 matrigel/PBS and implanted subcutaneously into NSG mice. Tumors were analyzed four weeks later. (**A**) Huh7 transfection with GPX4 containing plasmid was confirmed by western blotting, activity measurements and real-time RT-PCR. (**B**) Final tumor weight of tumors originating from control (vector) or GPx4 overexpressing Huh7 cells. $^{**}p < 0.01$ two-tailed *t*-test, $n = 14$ control tumors; $n = 16$ GPx4 overexpressing tumors. (**C**) Glutathione levels were analysed in GPx4 overexpressing and control tumors, $^{*}p < 0.05$ one-tailed t-test, n = 11-12 per group. (**D**) Thrombospondin 1 (THBS1) mRNA was analysed in tumor samples from GPx4 overexpressing and control Huh7 cells and normalized to GAPDH as a house keeping gene, $p = 0.059$ one-tailed ttest, n = 12-13 per group. (**E**) The same as (D) but C-reactive protein (CRP) mRNA was analysed. $^{**}p < 0.01$ two-tailed t-test, n = 12-13 per group. Vessel density (**F**) and vessel size distribution (**G**) in Huh7 xenograft tumors was assessed by IHC/tissue morphometric analysis using endomucin staining (n = 6-8 per group). (**H**) The percentage of KI-67 positive cells in tumor was analysed by IHC/tissue morphometric analysis ($p = 0.076$ Mann-Whitney one-tailed test, n = 7-8 per group).

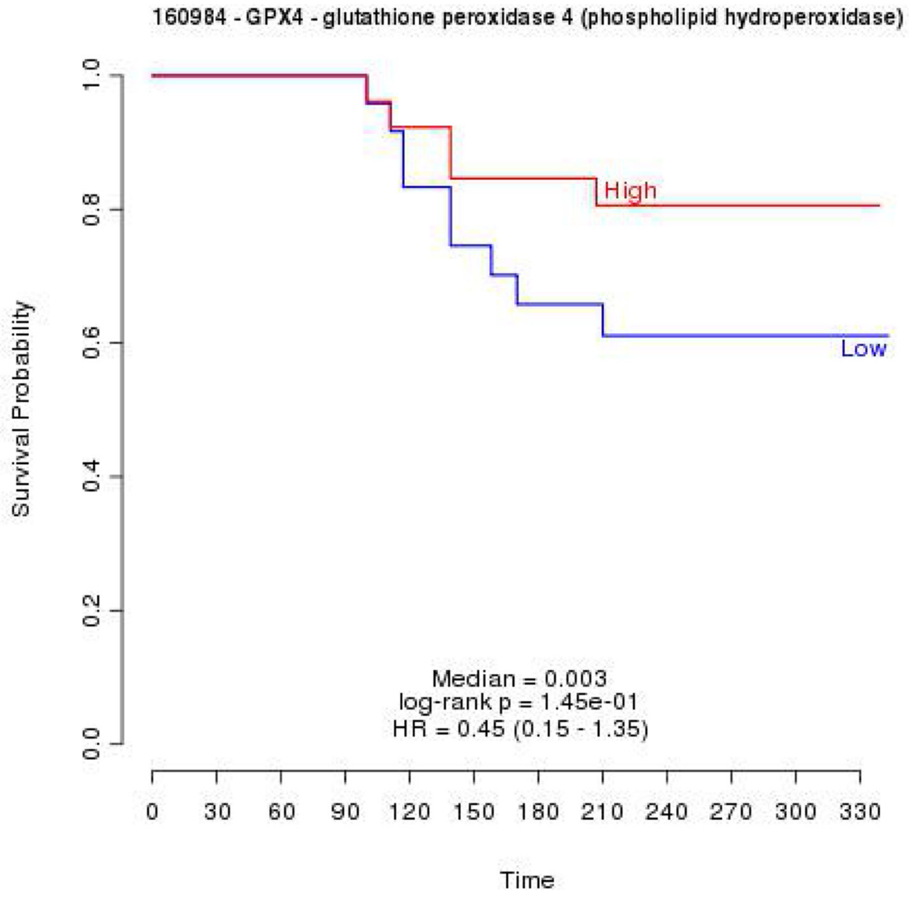

Supplementary Figure 6: Impact of GPx4 expression on survival of HCC patients with primary liver cancer. Microarray gene expression and survival data have been re-evaluated using PRECOG data base as described in Materials and Methods. Microarray gene expression data sets from the following publication have been re-evaluated: Ye QH, Qin LX, Forgues M, He P et al. Predicting hepatitis B virus-positive metastatic hepatocellular carcinomas using gene expression profiling and supervised machine learning. Nat Med 2003 Apr;9(4):416-23.

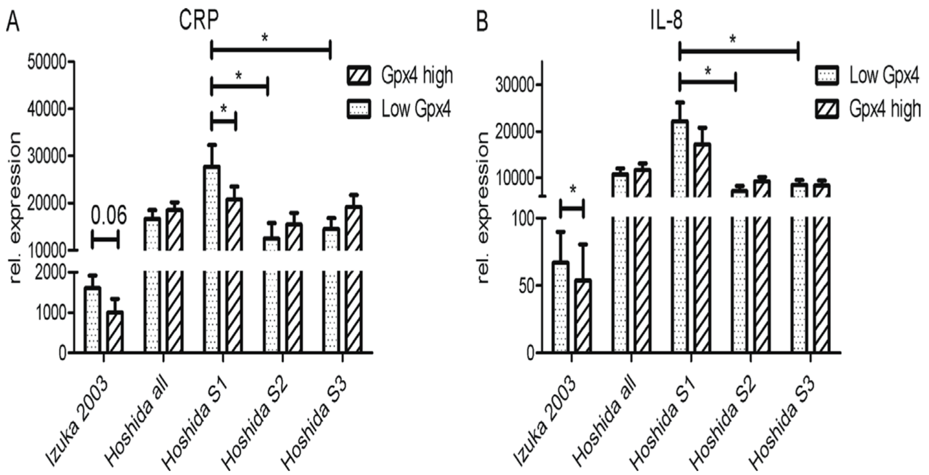

Supplementary Figure 7: Interactions of GPx4 with gene expression in human HCC patients. Re-evaluation of gene expression microarray data in tumor tissues from two cohorts of human HCC. The HCC cohort of Hoshida was also subdivided into molecular subclasses S1, S2 and S3. The patients were stratified according to the normalized GPx4 expression levels. Mean gene expression values were calculated for “Low GPx4” or “High GPx4” patient groups. Expression levels of C-reactive protein (CRP, A) and interleukin-8 (IL-8, B) are shown.

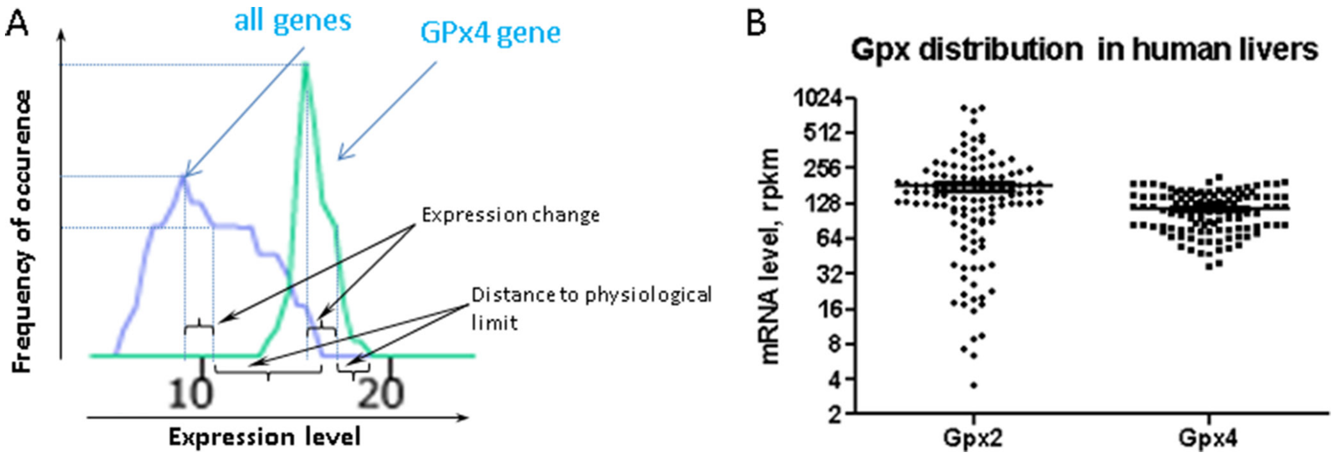

Supplementary Figure 8: Distribution width of GPx4 gene expression level in humans. (**A**) The global distribution width of GPx4 expression is narrow when compared with all genes (NCBI Aceview). For narrow distribution, even relatively small expression changes in GPx4 level may already bring it close to and even beyond the physiological limit as illustrated by a shorter distance to physiological limit. Wide distribution allows higher changes of expression levels until the physiological limit is reached. (**B**) Comparison of the distribution width of hepatic expression of GPx4 and GPx2 in humans. The data were extracted from the from the Human Protein Atlas, (www.proteinatlas.org; GTeX human liver dataset, *n* = 119).

Supplementary Table 1: Meta-analysis of associations between mRNA levels of GPx4 and other selenoproteins with cancer patient survival

Abbreviation	Selenoprotein	Unweighted Meta-Z of all cancers	Z-score primary Liver cancer[a]	Z-score Liver cancer[b]
GPx4	Glutathione peroxidase 4	-2,06	-2,26	-1,35
GPx1	Glutathione peroxidase 1	-1,2	-	0,06
GPx3	Glutathione peroxidase 3	-0,64	-	-0,96
GPx2	Glutathione peroxidase 2	-0,28	-0,92	0,87
GPx6	Glutathione peroxidase 6	0,12	-	-
TXNRD1	Thioredoxin reductase 1	4,08	0,25	-0,29
TXNRD2	Thioredoxin reductase 2	-2,26	-	-2,07
TXNRD3	Thioredoxin reductase 3	1,87	-	-
SELENBP1	Selenium-binding protein 1	-2,98	0,66	-0,66
SELM	Selenoprotein M	-3,94	-0,02	-
SELT	Selenoprotein T	-1,43	-	1,02
SELO	Selenoprotein O	-0,57	-	0,96
SELS	Selenoprotein S	0,31	-	1,59
SELV	Selenoprotein V	0,51	-	-
SELK	Selenoprotein K	-2,78	-	0,95
SELN	Selenoprotein N	0,05	-	-0,91
SELP	Selenoprotein P	-5,79	-	1,1
SELR	Selenoprotein R	3,3	-	-1,37
SEPHS1	Selenophosphate synthetase 1	0,08	-	0,4
SEPHS2	Selenophosphate synthetase 2	1,51	1,07	-2,05
SECISBP2	Selenocysteine insertion sequence binding protein 2	-3,5	-1,54	1,81
DIO1	Iodothyronine deiodinase 1	-2,82	-	-3,35
DIO2	Iodothyronine deiodinase 2	-0,89	0,13	1,06
DIO3	Iodothyronine deiodinase 3	-1,47	1,19	-1,5

The data have been obtained using PRECOG database 1. This database shows associations between microarray data on gene expression levels in tumors and survival of about 18000 patients. Z-scores are related to P values and encode both strength and direction of statistical associations. For example, $|z| > 1.96$ is equivalent to a two-sided $P < 0.05$. Positive z-scores reflect adverse association, negative z-scores favorable associations with survival 1.

a – data analysis from the cohort of Ye et al. 2; b - data analysis from the three independent cohorts 3-5

1. Gentles AJ, Newman AM, Liu CL, et al. The prognostic landscape of genes and infiltrating immune cells across human cancers. Nat Med. 2015; 21:938–945.
2. Ye QH, Qin LX, Forgues M, et al. Predicting hepatitis B virus-positive metastatic hepatocellular carcinomas using gene expression profiling and supervised machine learning. Nat Med. 2003; 9:416–23.
3. Hoshida Y, Villanueva A, Kobayashi M, et al. Gene expression in fixed tissues and outcome in hepatocellular carcinoma. N Engl J Med. 2008; 359:1995–2004.
4. Lee JS, Chu IS, Mikaelyan A, et al. Application of comparative functional genomics to identify best-fit mouse models to study human cancer. Nat Genet. 2004; 36:1306–11.
5. Lee JS, Heo J, Libbrecht L, et al. A novel prognostic subtype of human hepatocellular carcinoma derived from hepatic progenitor cells. Nat Med. 2006; 12:410–6.

Supplementary Table 2: Molecular networks differentially regulated in tumors of HCC patients with low and high GPx4 expression

ID	Molecules in Network	Score	Focus Molecules	Top Diseases and Functions
1	CCL19,CCL21,CD37,COL3A1,COL4A2,collagen,Collagen type IV,CTSS,DGKA,elastase,ERK1/2,Erm, Fc gamma receptor,GZMK,Ifn,IgG,IgG1,IGHG3,IGKC,Igm,IL12 (complex),IL7R,Immunoglobulin,JCHAIN,LAMA2,Laminin,MGP,MHC Class II (complex),PSMB10,Rap1,SELPLG,TAGLN,Tgf beta,	46	19	Cell-To-Cell Signaling and Interaction, Hematological System Development and Function, Antigen Presentation
2	Akt,Alp,Alpha catenin, CCL14, COL15A1, COL6A1,COL6A2,COL6A3,Collagen type I,Collagen type VI, Collagen(s), CXCL12, EFEMP1, estrogen receptor,F Actin,FHL2,GEM,HCLS1,Hsp27,IGFBP5,Integrin,LUM,Mlc,Mmp,MYH11,Notch,p70 S6k,PDGF BB,PI3K (family), PRCP, Raf, Rock, THBS2	34	16	Developmental Disorder, Hereditary Disorder, Skeletal and Muscular Disorders
3	ADCY,AMPK,ANXA1,Ap1,CD3,Cg,Ck2,Creb,CYP1B1,cytochrome C, ERK, F2R, Fibrinogen, FSH, G protein,G protein alphai,Hsp90,IFI16, Insulin, JUNB, Lh, Mapk, MEF2C, NFkB (complex),P38 MAPK,Pkc(s),PLC, Ras,RNA polymerase II,STAT,TCR, TMSB10/TMSB4X, TSH,UCP2,Vegf	14	8	Cardiovascular System Development and Function, Tissue Morphology, Cell Death and Survival
4	26s Proteasome, AQP1, C5, C7, C8, caspase, CCL19, CCL25, CCL27, CCL28, CD48, chemokine, CSRNP1, CXCL13, EMP, ERBB2, Focal adhesion kinase, FRS3, glycosylphosphatidylinositol, IL6, IL31, IL1B, Interferon alpha, Jnk, LUM, mir-138, OSBPL1A, p85 (pik3r),PI3K (complex),PRELP,Rac	13	7	Cell-mediated Immune Response, Cellular Movement, Hematological System Development and Function

Supplementary Table 3: Immune cell profiling in tumor tissues of HCC patients with low and high expression of GPx4

Immune cell types	Mean amount in patients with low GPx4	Stdev for low GPx4 patients	Mean amount in patients with high Gpx4	Stdev for high GPx4 patients	Fold change high/low GPx4	ttest
B cells naive	0,043	0,043	0,050	0,037	1,160	0,176
B cells memory	0,028	0,035	0,011	0,020	**0,407**	**0,001**
Plasma cells	0,024	0,039	0,007	0,026	**0,269**	**0,003**
T cells CD8	0,121	0,063	0,129	0,079	1,070	0,255
T cells CD4 naive	0,000	0,000	0,000	0,000	-	-
T cells CD4 memory resting	0,216	0,078	0,214	0,096	0,990	0,445
T cells CD4 memory activated	0,036	0,035	0,026	0,027	**0,735**	**0,055**
T cells follicular helper	0,090	0,048	0,050	0,046	**0,553**	**0,000**
T cells regulatory (Tregs)	0,007	0,012	0,021	0,029	**3,031**	**0,000**
T cells gamma delta	0,010	0,021	0,000	0,000	**0,000**	**0,000**
NK cells resting	0,006	0,016	0,015	0,028	**2,401**	**0,012**
NK cells activated	0,015	0,024	0,042	0,044	**2,736**	**0,000**
Monocytes	0,006	0,009	0,007	0,011	1,067	0,408
Macrophages M0	0,002	0,005	0,026	0,035	**13,491**	**0,000**
Macrophages M1	0,081	0,028	0,110	0,037	**1,366**	**0,000**
Macrophages M2	0,194	0,063	0,143	0,079	**0,737**	**0,000**
Dendritic cells resting	0,007	0,014	0,022	0,030	**2,952**	**0,000**
Dendritic cells activated	0,003	0,006	0,000	0,001	**0,052**	**0,003**
Mast cells resting	0,035	0,043	0,079	0,054	**2,264**	**0,000**
Mast cells activated	0,015	0,026	0,003	0,017	**0,188**	**0,002**
Eosinophils	0,013	0,020	0,000	0,003	**0,032**	**0,000**
Neutrophils	0,047	0,036	0,045	0,028	0,965	0,392

Supplementary Table 4: CIBERSORT neutrophil counts in HCC patients with low and high intratumoral IL-8 expression

Patient group	*n*	Neutrophil fraction, % of all immune cells	
		Mean + SD	95% confidential interval
Low IL-8	153	3.7 + 3.1	3.2 – 4.1
High IL-8	152	5.2 + 3.6**	4.6 – 5.8

**$P < 0.0001$ two-tailed Mann-Whitney test between low and high IL-8 groups.
Neutrophil fraction was calculated for each patient using CIBERSORT approach (s. Materials and Methods section).

Supplementary Table 5: Estimated contribution of immune cells populations to EMR1/F480 levels in patients from "low GPx4" and "high GPx4" groups

Gene symbol	EMR1, rel expression	Mean % of cells in "low Gpx4" group	contribution of cell types to EMR1/ F480 staining in "low Gpx4"	Mean % of cells in "high Gpx4" group	contribution of cell types to EMR1/F480 staining in "high Gpx4"	delta F480
Eosinophils	16330,7	0,013	212,3	0	0	212,3
Macrophages M2	404,4	0,194	78,5	0,143	57,8	20,6
T cells follicular helper	182,9	0,09	16,5	0,05	9,1	7,3
B cells memory	383,1	0,028	10,7	0,011	4,2	6,5
T cells gamma delta	448,0	0,01	4,5	0	0,0	4,5
T cells CD4 memory activated	400,2	0,036	14,4	0,026	10,4	4,0
Plasma cells	194,4	0,024	4,7	0,007	1,4	3,3
Mast cells activated	248,2	0,015	3,7	0,003	0,7	3,0
Neutrophils	1253,6	0,047	58,9	0,045	56,4	2,5
Dendritic cells activated	237,4	0,003	0,7	0	0,0	0,7
T cells CD4 memory resting	312,8	0,216	67,6	0,214	66,9	0,6
T cells CD4 naive	428,1	0	0,0	0	0,0	0,0
T cells CD8	195,9	0,121	23,7	0,129	25,3	-1,6
Monocytes	1815,5	0,006	10,9	0,007	12,7	-1,8
Dendritic cells resting	178,7	0,007	1,3	0,022	3,9	-2,7
NK cells resting	348,1	0,006	2,1	0,015	5,2	-3,1
B cells naive	499,0	0,043	21,5	0,05	25,0	-3,5
T cells regulatory (Tregs)	335,8	0,007	2,4	0,021	7,1	-4,7
Macrophages M0	203,3	0,002	0,4	0,026	5,3	-4,9
NK cells activated	188,5	0,015	2,8	0,042	7,9	-5,1
Mast cells resting	287,4	0,035	10,1	0,079	22,7	-12,6
Macrophages M1	3528,2	0,081	285,8	0,11	388,1	-102,3
		total EMR1/F4/80 staining intensity	833,2		710,2	
